# Metafabrics for cooling under a scorching sun

**DOI:** 10.1038/s41377-021-00669-5

**Published:** 2021-10-27

**Authors:** Shuang Zhang

**Affiliations:** 1 Department of Physics, The University of Hong Kong, Pokfulam Road, Hong Kong, China; 2 Department of Electrical and Electronic Engineering, The University of Hong Kong, Pokfulam Road, Hong Kong, China

**Keywords:** Metamaterials, Solar energy and photovoltaic technology

## Abstract

Engineering the spectral response of composite materials in a broad range from ultraviolet to infrared can lead to a significant passive cooling functionality. This principle is applied to the design of a novel type of metafabric for cooling the human body under direct sunlight. Besides cooling effect, the metafabric features other merits including superior mechanical and wetting properties.

Certain living creatures have developed the capability to control their body temperature in scorching weather conditions. For example, human can release heat through sweating, with heat taken away via the evaporation process. Interestingly, some creatures can keep themselves cool through a process called radiative cooling, such as the Saharan silver ants. They can maintain a lower body temperature than the ambient temperature, protecting themselves from the harsh hot desert climate. This cooling effect arises from a unique optical property of their skin—strong reflection of UV and visible light from the sun (the reason they appear silver color), and a strong absorption at infrared window from 8 to 12 μm^1^. Absorption and radiation are reciprocal processes, hence an object with strong absorption must be a good emitter within the same wavelengths range. Since the radiation falling in this bandwidth can escape the atmosphere, the heat can be delivered to the outer space, which has an average background temperature of 2.7 kelvins (−270.45 °C). This cooling can work even under direct sunlight, due to the high efficiency in reflecting off UV, visible, and near-infrared (NIR) light (Fig. 1).

**Fig. 1:**
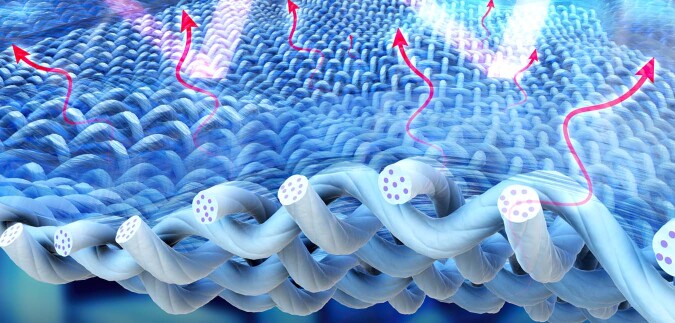
A schematic of the metafabric for achieving passive cooling. The metafabric can absorb light in the mid-infrared and reflect light of shorter wavelengths (near-infrared, visible and ultraviolet)

Over the past decade, tremendous progress has been made in the field of passive radiative cooling, significantly reducing the cost and increasing the applicability of cooling devices in various fields. One of the earliest demonstrations of passive radiative cooling was made by Prof. Shanhui Fan’s group from Stanford University^2^. In their pioneering work, multiple layers of dielectric materials including silicon dioxide, hafnium dioxide, and silver with a total thickness of nearly 2 μm were designed to achieve the radiative cooling effect. The cooling layers were attached to solar cells to reduce the heating effect for a better performance. Despite the superior performance in cooling, the fabrication of the device was limited to relatively small area and suffered from high cost. Several years later, a new approach was invented for mass production of radiative cooling films at a much lowered cost^3^. The radiative cooling film is essentially a plastic film containing densely embedded silica microspheres. The silica spheres can strongly absorb light at mid-infrared owing to the Mie resonant effect. Thus, the distribution of the sizes of the microsphere can be judiciously engineered to exhibit a broadband absorption and emission at 8–12 μm window. The film could be manufactured at low cost through roll-to-roll manufacturing processes. Thus this new development paved way toward practical applications such as cooling of building and, etc. During the past few years, many interesting ideas have emerged for achieving low cost cooling materials in various forms, such as paints^4,5^, polymers^6^, structural materials^7^, and textiles^8^, aiming for applications in diverse areas. However, the textiles developed for cooling has been too thin to be suitable for clothing applications.

Reporting in Science in 2021, a team led by Prof. Guangming Tao from Huazhong University of Science and Technology and Prof. Yaoguang Ma from Zhejiang University demonstrated the first wearable cooling fabric, which not only exhibits very good cooling performance, but also features superior structural and wetting properties, thus representing a very promising and pragmatic approach for body temperature management^9^. The metafabric consists of several key ingredients structured in a hierarchical morphology, with each ingredient accounting for a particular functionality. The combination of these ingredients reaches desirable spectral response in a very broad bandwidth from DUV to long Mid-infrared, hence resulting in efficient cooling under direct sunlight. Specifically, the metafabric consists of a titanium oxide–polylactic acid (TiO_2_–PLA) composite woven textile laminated with a thin polytetrafluoro-ethylene (PTFE) layer, which is a commonly used material for the clothing industry. The PTFE layer consists of nanoparticles of different sizes in the range of a few hundred nanometers. With suitably designed size range, the PTFE layer is capable of strongly reflecting UV light from the sun. The distribution of the TiO_2_ particles is designed in such a way that the visible light is scattered away. Thus the PTFE layer and TiO_2_ combined together ensures that sunlight does not penetrate through or generate heat in the fabric. The main function of the PLA substrate in TiO_2_–PLA fibers is to carry heat away by emitting thermal radiation in the infrared range. The PLA has a bond that can resonate at mid-IR, which provide the efficient thermal emission functionality. Thus, the combination of the three materials with distinct and complimentary functionalities provides the desired optical properties for radiative cooling.

The radiative cooling performance of the metafabric was tested on a human skin simulator under direct sunlight. It was shown that the metafabric could reach a cooling capability 5.0°–10 °C lower than that of the common wearable materials such as cotton and linen. In a control experiment, a volunteer wore a homemade vest made by sewing a commercial cotton fabric and a metafabric together and sat under direct sunlight for one hour. A significant temperature difference of 3.4 °C between the two sides of the vest was detected, verifying the efficacy of the passive cooling metafabric.

Besides its performance in cooling, the metafabric also shows impressive structural and surface effects. Specifically, the metafabric was designed to be hydrophobic on one side and hydrophilic on the other. With the hydrophobic side facing outside, the metafabric can repel water and stay dry in a rainy day. The fabric is highly stretchable, not showing any signs of damage under stretching up to 20%. With its superior performance in radiative cooling performance, breathability, and wearing comfort, a wide range of applications can be envisioned for the designed metafabrics, including clothing, tents, and car covers.
